# Targeted Next-Generation Sequencing of Circulating Tumor DNA, Bone Marrow, and Peripheral Blood Mononuclear Cells in Pediatric AML

**DOI:** 10.3389/fonc.2021.666470

**Published:** 2021-07-29

**Authors:** Min Ruan, Lipeng Liu, Benquan Qi, Xiaoyan Chen, Lixian Chang, Aoli Zhang, Fang Liu, Shuchun Wang, Xiaoming Liu, Xiaojuan Chen, Li Zhang, Ye Guo, Yao Zou, Yingchi Zhang, Yumei Chen, LiXia Liu, Shanbo Cao, Feng Lou, Chengcheng Wang, Xiaofan Zhu

**Affiliations:** ^1^Division of Pediatric Blood Diseases Center, State Key Laboratory of Experimental Hematology, National Clinical Research Center for Blood Diseases, Institute of Hematology & Blood Diseases Hospital, Chinese Academy of Medical Sciences & Peking Union Medical College, Tianjin, China; ^2^Executive President Office, Acornmed Biotechnology Co., Ltd., Beijing, China; ^3^Medical Department, Acornmed Biotechnology Co., Ltd., Beijing, China

**Keywords:** acute myeloid leukemia, targeted next-generation sequencing, circulating tumor DNA, mutation (genetics), pediatric

## Abstract

**Background:**

The aim of the study was to validate the diagnostic role of circulating tumor DNA (ctDNA) in genetics aberration on the basis of next-generation sequencing (NGS) in pediatric acute myeloid leukemia (AML).

**Methods:**

Bone marrow (BM) and peripheral blood (PB) were collected from 20 AML children at the time of initial diagnosis, and a ctDNA sample was isolated from PB. Detection of mutation was performed on ctDNA, BM, and peripheral blood mononuclear cell (PBMC) by NGS based on a 185-gene panel.

**Results:**

Among 185 genes sequenced by the NGS platform, a total of 82 abnormal genes were identified in 20 patients. Among them, 61 genes (74.39%) were detected in ctDNA, PBMC, and BM samples, while 11 (13.41%) genes were found only in ctDNA and 4 (4.88%) were detected only in the BM sample, and 2 (2.44%) were detected only in PBMC. A total of 239 mutations were detected in three samples, while 209 in ctDNA, 180 in bone marrow, and 184 in PBMC. One hundred sixty-four mutations in ctDNA were shared by matched BM samples, and the median variant allelic frequency (VAF) of these mutations was 41.34% (range, 0.55% to 99.96%) and 44.36% (range, 0.56% to 99.98%) in bone marrow and ctDNA. It was found that 65.79% (75/114) of mutations with clinical significance were detected in three samples, with 9 mutations detected both in ctDNA and BM, and 2 mutations detected both in PBMC and BM. The consistency of mutations with clinical significance between ctDNA and BM was 77.06% (84/109). Among the 84 mutations with clinical significance detected in both sources, the concordance of VAF assessment by both methods was high (R^2^ = 0.895).

**Conclusion:**

This study demonstrates that ctDNA was a reliable sample in pediatric AML and can be used for mutation detection. Consistency analysis showed that ctDNA can mirror the genomic information from BM. In addition, a subset of mutations was exclusively detected in ctDNA. These data support the fact that monitoring ctDNA with next-generation sequencing-based assays can provide more information about gene mutations to guide precision treatment in pediatric AML.

## Introduction

Acute myeloid leukemia (AML), which accounts for 25% of childhood acute leukemia, is a rapidly progressing hematopoietic malignancy characterized by the differentiation block and aberrant proliferation of leukemic blasts (1). In pediatric AML patients, the achieved 5-year overall survival (OS) is 60–70%,while the event-free survival (EFS) is 50% (2). With the development of molecular biology technology, the molecular landscape of pediatric AML becomes clearer (3, 4). Mutations in FLT3, TP53, NPM1, CEBPA, RUNX1, and ASXL1, which are common in AML children, have received more and more attention. And the clinical significance of new mutations, such as STAG2, RAD21, SRSF2, and U2AF1, have been gradually clarified. These diverse genomic molecular markers reflect the heterogeneity of AML, and accurate molecular profiling in AML is important for risk stratification and selection of targeted therapies (5).

Circulating tumor DNA (ctDNA), which is contained in circulating-free cell DNA (cfDNA) and released by necrosis or apoptosis tumor cell, allows for noninvasive peripheral blood sampling of cancer-associated mutations (6–9). When compared with other samples, ctDNA is more like a genomic library of different tumor cells and can mirror the heterogeneity of AML; moreover, ctDNA has a relatively short half-life, which may better reflect the latest status of the disease (10–12).

Nowadays, noninvasive detection of mutations by ctDNA was widely used in various solid tumors, but its role in hematological malignancies is still not clear. The current “gold standard” for molecular testing in pediatric AML is from bone marrow (BM) aspirate DNA. However, BM aspiration is an invasive procedure, which severely limited its application in clinical research. To date, there are very limited studies on the potential role of ctDNA, as a relatively non-invasive source, in monitoring leukemia-associated mutations and providing prognostic information in patients with hematologic malignancies (13, 14). Furthermore, it is still unknown whether ctDNA can fully supplant BM assessment for molecular profiling in pediatric AML.

Therefore, we aim to validate the diagnostic role of ctDNA in molecular profiles in pediatric AML patients, when compared with hybrid capture-targeted next-generation sequencing of BM, peripheral blood mononuclear cell (PBMC).

## Materials and Methods

### Patients and Patient Specimens

For this prospective analysis, the source population included 20 children (age < 18 years) with AML at the Division of Pediatric Blood Diseases Center in Institute of Hematology and Blood Diseases Hospital, Chinese Academy of Medical Sciences & Peking Union Medical College. BM and PBMC were collected for diagnostic purposes from all enrolled patients (excluding Down syndrome or acute promyelocytic leukemia, secondary AML), while ctDNA was isolated from the PB samples. The data collected included information regarding age, sex, peripheral blood white blood cell counts (WBC), blast percentages in BM and PBMC, chromosome karyotypes, and gene mutation signatures.

The study design and methods complied with the Declaration of Helsinki and were approved by the Ethics Committee and Institutional Review Board of Institute of Hematology and Blood Diseases Hospital, Chinese Academy of Medical Sciences & Peking Union Medical College. Informed consent was obtained from all subjects. The raw sequence data reported in this paper have been deposited in the Genome Sequence Archive (Genomics, Proteomics & Bioinformatics 2017) in National Genomics Data Center (Nucleic Acids Res 2021), China National Center for Bioinformation/Beijing Institute of Genomics, Chinese Academy of Sciences, under accession number HRA000912, which are publicly accessible at https://ngdc.cncb.ac.cn/gsa-human.

### Next-Generation Sequencing and Mutation Analysis

cfDNAs were extracted by a customized QIAamp Circulating Nucleic Acid kit (Qiagen GmbH) from 20 patient’s PB samples at diagnosis, while DNA were extracted by a customized Genomic DNA kit (Qiagen GmbH) from the patient’s BM and PBMC samples. Gene library amplification was based on a KAPA Hyper Prep Kit. The gene panel from Acornmed Biotechnology was used to capture the target region. Detailed sequencing information is provided in **Table S1**.

Multiplexed libraries were sequenced with Illumina Novaseq and then analyzed for data including Sequencing mapping, coverage and quality assessment, Insertion/Deletion detection, annotation for sequence mutations: Average raw sequencing depth on target per sample ≥10000x(ctDNA)≥1000x (DNA), Allele mutation frequency ≥0.5% for single Nucleotide variation and insertion or deletion, respectively. All reads were filtered by high Mapping quality (≥30) and Base quality (≥30). The mutant reads were supported by positive and negative strands. Reads were aligned to the human genome using the Burrows-Wheeler Alignment tool (BWA, version 0.7.12). PCR duplicates were marked by the MarkDuplicates tool in Picard. IndelRealigner and BaseRecalibrator on Genome Analysis Toolkit (GATK; version 3.8) were used for the realignment and recalibration of the BWA alignment results, respectively. Mutect2 was used for identifying SNV and INDEL. We obtained candidate variations through background database filtering of normal samples. Pindel was used for detecting FLT3-ITD. FLT3-ITD quantitative analysis was performed by in-house tools based on machine learning development. All the variants were annotated by the ANNOVAR software using some resources, including 1000G projects, COSMIC, SIFT, and Polyphen. Our gene panel was mainly from NCCN guidelines, EMSO guidelines, authoritative databases, and literature reports of hematologic tumors.

### Statistical Analysis

Patient characteristics were summarized using median (range) for continuous variables and frequencies (percentages) for categorical variables. The Fisher exact test was used to test the association between two categorical variables. Concordance of BM and ctDNA and PBMC results were assessed using Pearson correlation analysis. *P* values<0.05 were considered significant. All statistical tests were performed using SPSS 24.0 (IBM Corporation).

## Results

### Baseline Characteristics

Twenty patients with newly diagnosed AML were evaluated. The baseline characteristics of the study cohort are shown in **Table 1**. Four patients (20%) had absolute PB blast count<1×10^9^/L, and one patient had no peripheral blood circulating blasts. ctDNA, PBMC, and BM targeted sequencing were performed in all 20 patients at diagnosis simultaneously.

**Table 1:** Baseline characteristics of the study population.

Characteristic	Median [range] or n (%)
Sex	
Male	7 (35)
Female	13 (65)
Age, years	11 (1-15)
White blood cells, ×10^9^/L	25.82 (1.54-182.5)
Peripheral blood blasts, ×10^9^/L	12.41 (0-124.1)
Peripheral blood blasts, %	36 (0-98.0)
Bone marrow blasts, %	66.5 (21.0-90.0)
Cytogenetics	
t (8;21)	6 (30)
inv (16)	1 (5)
t (6;11)	1 (5)
+8	2 (10)
-7	1 (5)
Complex karyotype	1 (5)
Normal karyotype	8 (40)
FAB type	
M1	2 (10)
M2	8 (40)
M4	6 (30)
M5	3 (15)
M7	1 (5)

### Detection of Molecular Profiles by Three Methods

The molecular profiles of all patients were detected by target-Next-generation Sequencing (t-NGS) (Acornmed Biotechnology Co., Ltd.), which covers the most frequent mutations in 137 genes in AML patients, *via* ctDNA, BM, and PBMC samples (**Table S1**). The sequencing depths of the three samples were all greater than 2000 X, namely 3460X (1837X-4270X) in ctDNA, 2530X (1633X-2862X) in BM, and 2324X (1208X-3720X) in PBMC.

**Figure 1:**
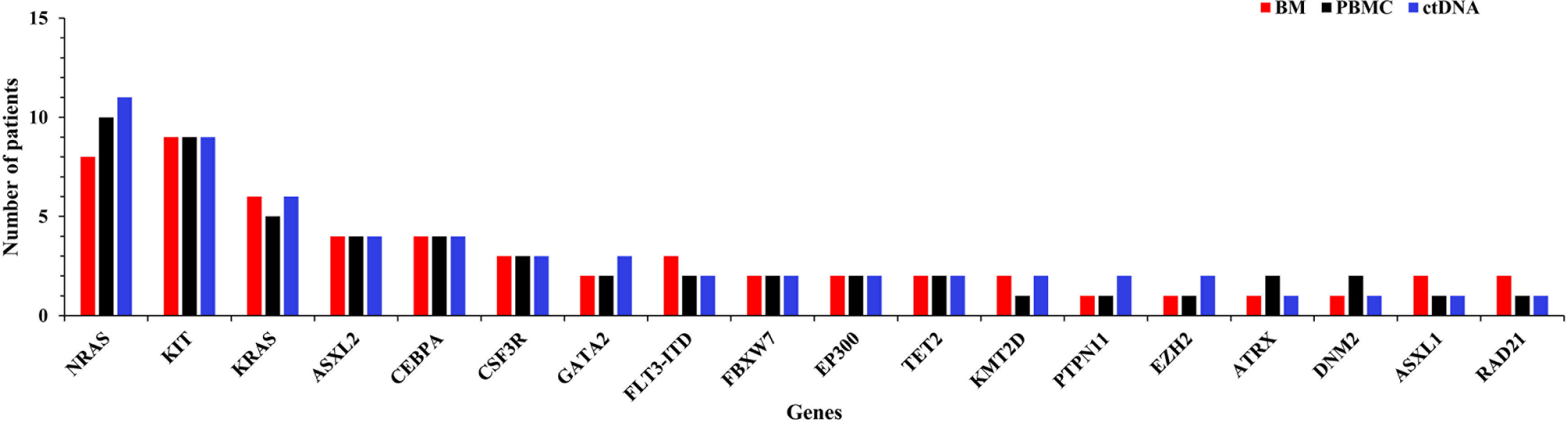
Frequency of AML-related mutated genes as detected by targeted sequencing of bone marrow (BM), peripheral blood mononuclear cell (PBMC), and circulating tumor DNA (ctDNA) samples.

A total of 82 abnormal genes were identified in 20 included patients. Among them, 61 genes (74.39%) were detected in ctDNA, PBMC, and BM samples, while 11 (13.41%) genes were found only in ctDNA, 4 (4.88%) were detected only in BM sample, and 2 (2.44%) were detected only in PBMC. There were 18 genes with mutation frequency ≥10% in this study, and 38.89% (7/18) of them were identified by both methods. Eleven genes, namely NRAS (8, 10 and 11), KIT (9, 9 and 10), KRAS (6, 5 and 6), ASXL2 (4, 4 and 4), CEBPA (4, 4 and 4), CSF3R (3, 3 and 3), GATA2 (2, 2 and 3), FLT3-ITD (3, 2 and 2), FBXW7 (2, 2 and 2), EP300 (2, 2 and 2), and TET2 (2, 2 and 2), were with mutation frequency ≥10% of BM, PBMC, and ctDNA sequencing (**Figure 1**).

What’s more, a total of 239 mutation forms in 80 abnormal genes were detected in three samples; among all mutations, there were 180 in BM, 203 in ctDNA, and 184 in PBMC. Variant allelic frequencies (VAFs) of 180 mutations in BM were from 0.52% to 99.96% (median 37.97%), including 131 single nucleotide variations (SNVs) and 49 indels. For each patient, the median number of mutation is 9 (3-14); specifically, the number of SNVs was 6.5 (3 to 12) and that of indels was 2.0 (0-12). When compared with the BM sample, more mutations were found in ctDNA with VAFs from 0.50% to 99.98% (median 34.49%), including 150 SNVs and 53 indels. For each patient, the median number of the mutation was 10 (3 to 20), and 7.0 (3 to 20) SNVs and 3.0 (0 to 11) indels were identified. A total of 184 mutations were detected in PBMC with VAFs from 0.51% to 100.00% (median 34.72%), including 140 SNVs and 44 indels. For each patient, the number of mutations ranged from 3 to 20 (median: 8.5), and the median number of SNVs was 6.5 (3 to 12) and that of indels was 2.0 (0 to 8).

### Concordance of Mutation Detection in ctDNA and BM

**Figure 2:**
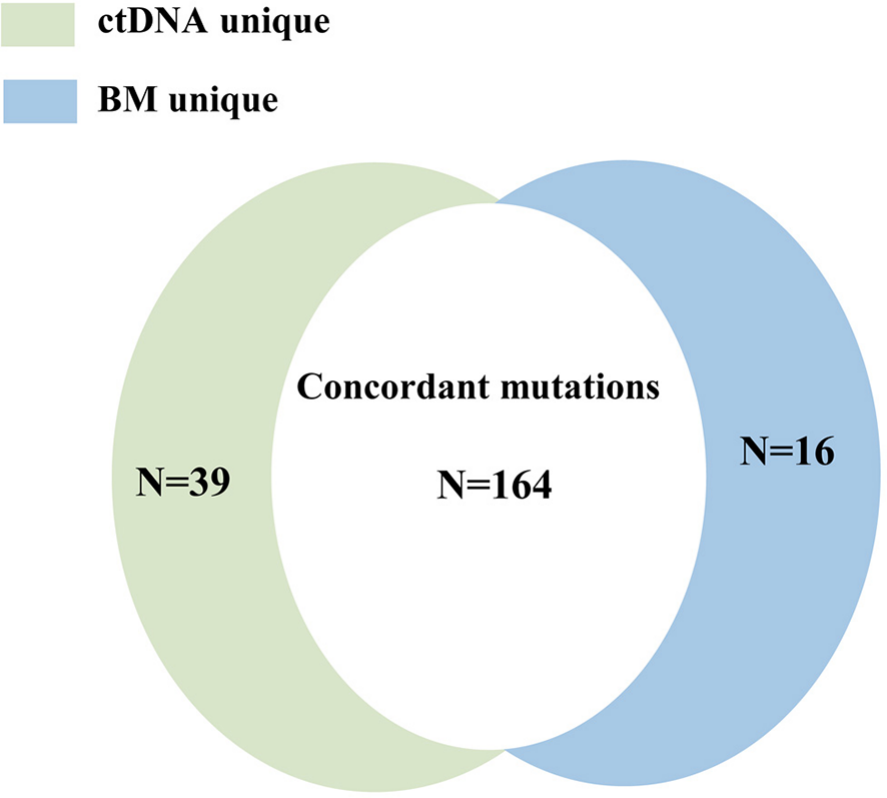
The concordance of the number of mutation detected in BM and ctDNA.

**Figure 3:**
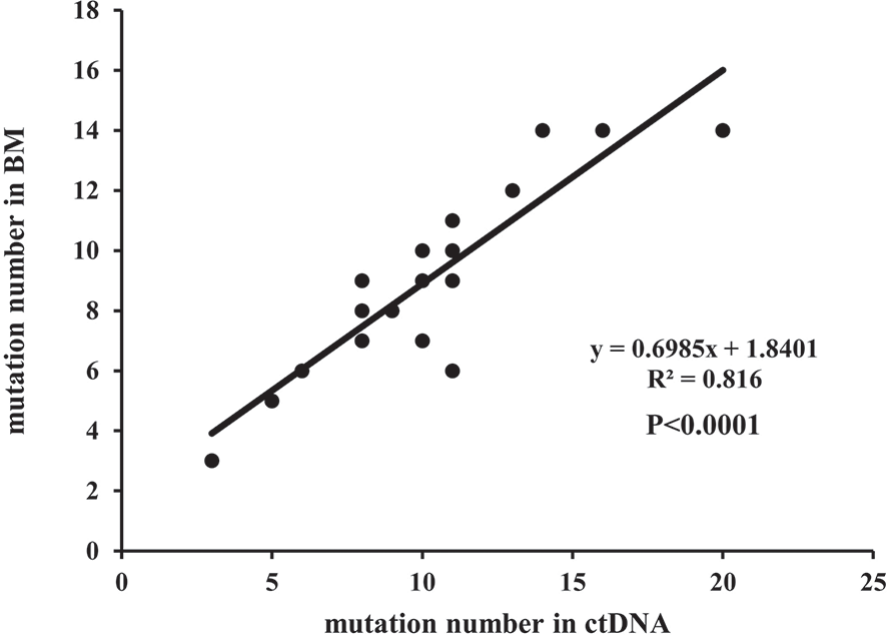
The correlation of the number of mutations in a separate patient detected in BM and ctDNA.

A total of 219 mutations were identified in BM and ctDNA, while 164 mutations (74.88%) were detected both in ctDNA and BM (**Figure 2**), including 121 SNVs (75.63%) and 43 indels (72.88%). Five patients (UPN3, UPN7, UPN14, UPN15, and UPN16) were with the same mutation sites according to BM and ctDNA sequencing (**Figure S1**). The median absolute blast count in PB of these patients was 19.33 ×10 ^9^/L (0.89 to 43.80 ×10 ^9^/L), which was higher than other patients. There was only one patient (UPN6) with more mutation sites detected in BM than ctDNA (**Figure S1**), and the absolute blast count in PB was 1.23 ×10 ^9^/L. There were 11 patients with more mutation sites detected in ctDNA than BM (**Figure S1**); the median absolute blast count of PB in these patients was 11.51 ×10 ^9^/L, which was similar to others. Although the number of mutations was similar in three patients (UPN9, UPN10, and UPN13) on the basis of BM and ctDNA, all sites of mutation were completely different (**Figure S1**). For each patient, 8 (2 to 14) mutations were detected in both samples, with high concordance of the number of mutation assessment by both methods (R^2^ = 0.816, *P <*0.0001; **Figure 3**).

**Figure 4:**
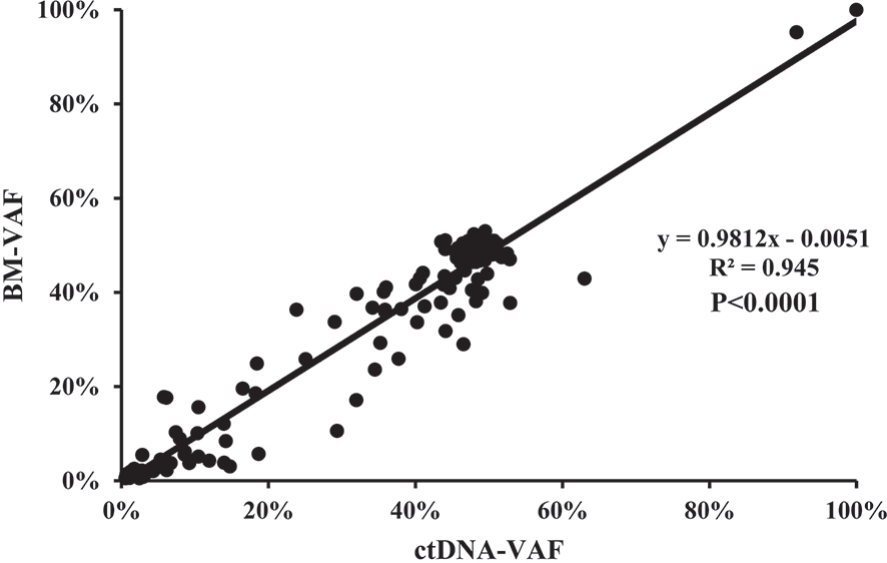
The correlation of the variant allelic frequencies (VAFs) in the same mutation site detected in BM and ctDNA among the 164 individual mutations detected by both assays.

**Figure 5:**
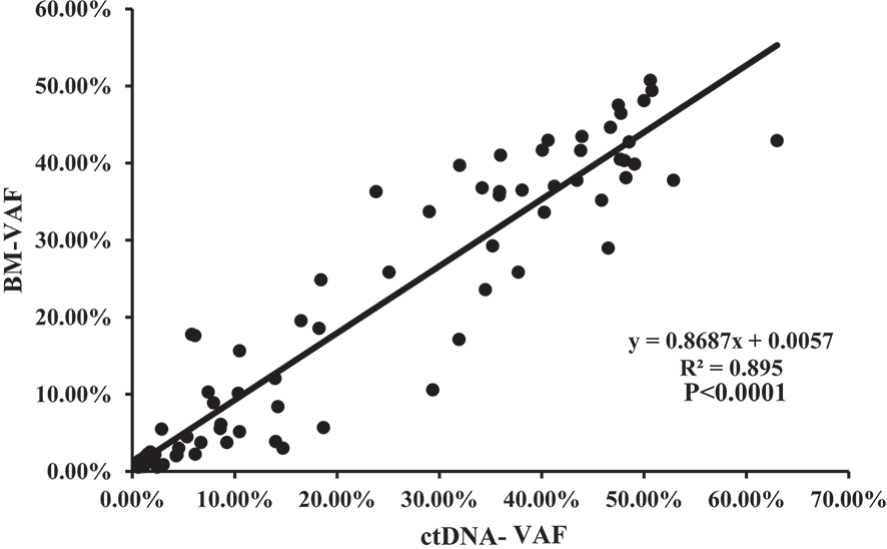
The correlation of the VAFs in the same mutation site detected in BM and ctDNA among the 84 mutations with clinical significance detected by both assays.

The median VAF of the 164 individual mutations detected by both assays was 41.34% (range, 0.55% to 99.96%) and 44.36% (range, 0.56% to 99.98%), and the concordance was high in all mutations (R^2^ = 0.945; *P*<0.0001, **Figure 4**), both in SNVs (R^2^ = 0.948; *P*<0.0001, **Figure S2A**) and indels (R^2^ = 0.934; *P*<0.0001, **Figure S2B**). The median VAFs of 16 mutations only detected by BM was 1.22% (0.52% to 14.63%), while it was 0.93% (0.50% to 21.14%) in 39 mutations tested by ctDNA only, and the VAFs were <1% in most of these mutations (**Figure S3**). In view of this, small subclonal populations with lower VAFs<1% were more likely to be missed. A total of 37 mutated genes with clinical significance were detected in all patients, involving 109 mutation sites (93 in BM and 100 in ctDNA). A total of 84 (77.06%) mutations were detected in both samples with high concordance of VAF assessment (R^2^ = 0.895, *P*<0.0001; **Figure 5**).

### Concordance of Mutation Detection in BM and PBMC

**Figure 6:**
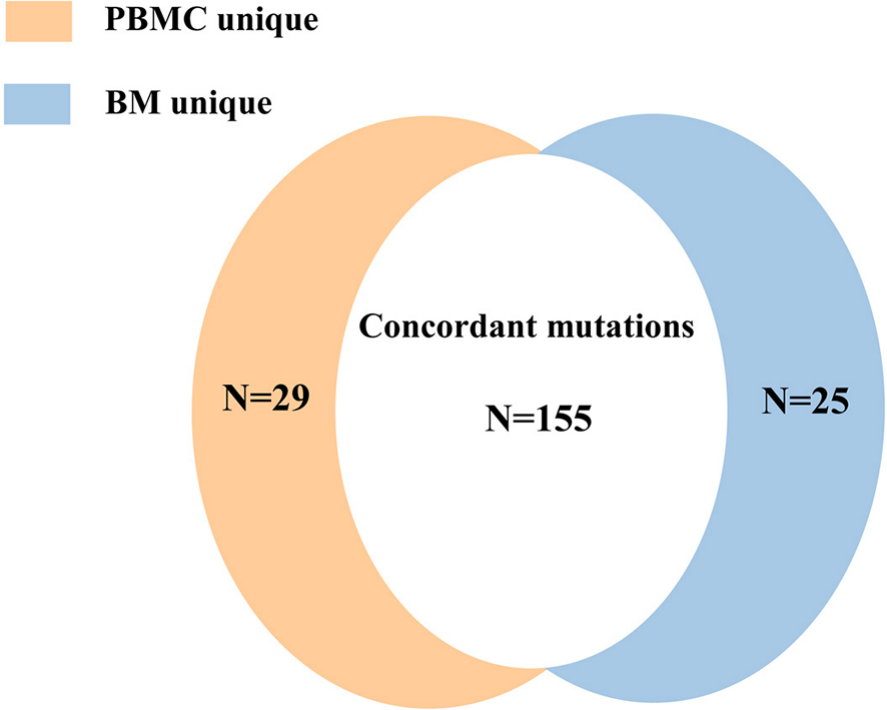
The concordance of the number of mutation detected in BM and PBMC.

**Figure 7:**
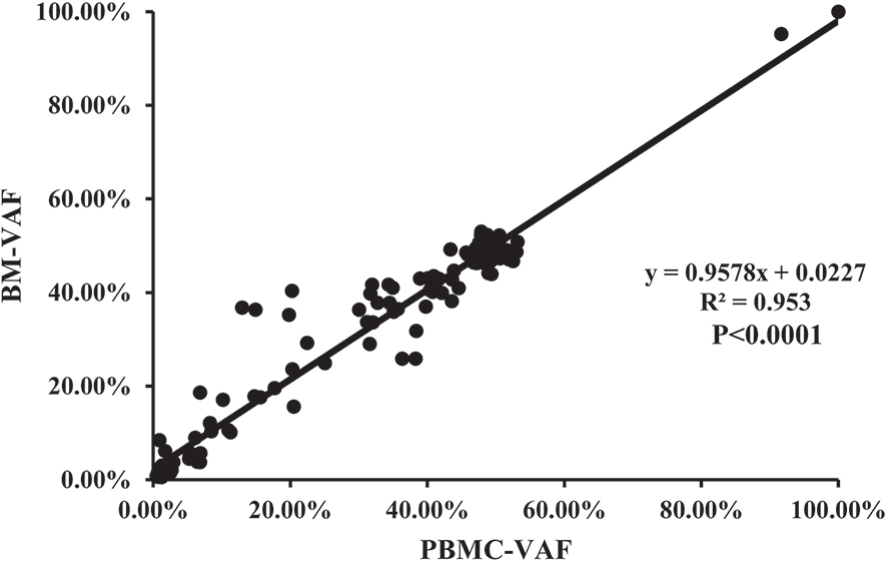
The correlation of the VAFs in the same mutation site detected in BM and PBMC among the 155 individual mutations detected by both assays.

Mutations were detected in PBMC samples of all patients at diagnosis in comparison to BM. A total of 155 mutations (74.16%) were found both in PBMC and BM (**Figure 6**), with high concordance of VAF assessment (R^2^ = 0.953, *P*<0.0001; **Figure 7**).

### Analysis of Mutations With Clinical Significance Detected in BM, ctDNA, and PBMC

**Figure 8:**
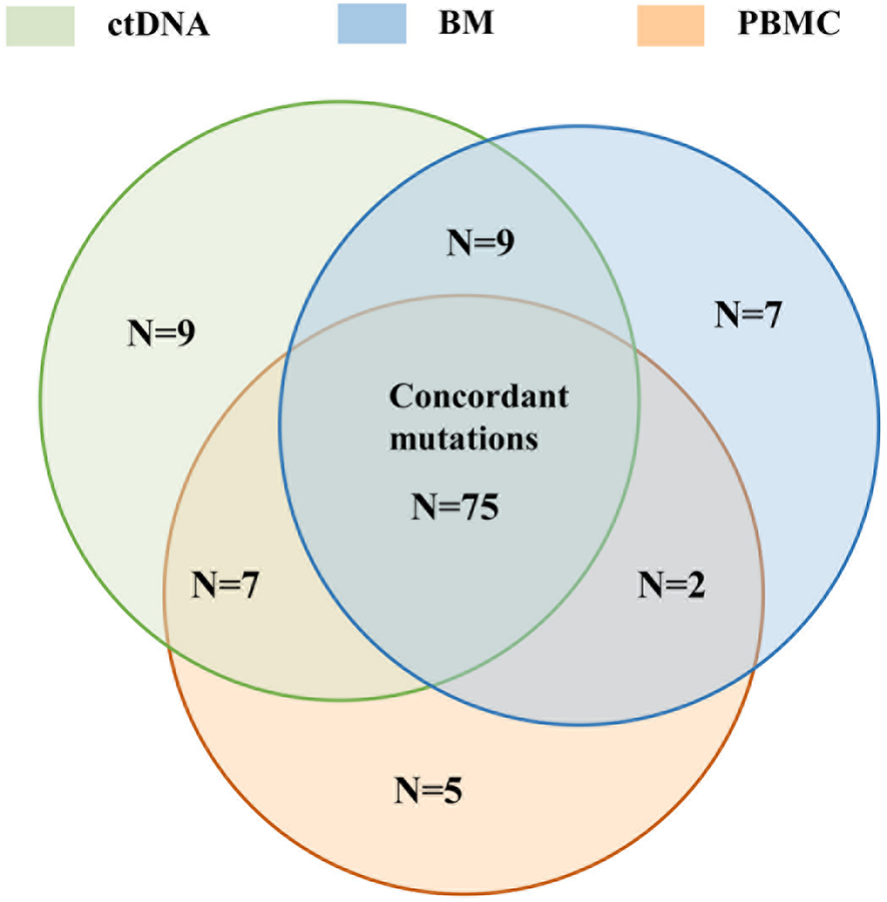
The concordance of the number of mutation detected in BM, ctDNA, and PBMC with clinical significance detected by all assays.

It was found that 65.79% (75/114) of mutations with clinical significance were detected in three samples, with 9 mutations detected both in ctDNA and BM, and 2 mutations detected both in PBMC and BM (**Figure 8**). The same mutated genes with clinical significance were detected in three samples in five patients (UPN4, UPN7, UPN12, UPN16, UPN20) with high concordance of VAF assessment (**Figure S4**). In addition to three sample co-detecting mutations, the remaining mutations were mostly detected in BM or ctDNA (**Figure S5**). These results suggested that PBMC cannot accurately reflect the mutations of bone marrow.

### Comparison of Bone Marrow and ctDNA in Minimal Residual Disease Assessment in AML Patients

**Figure 9:**
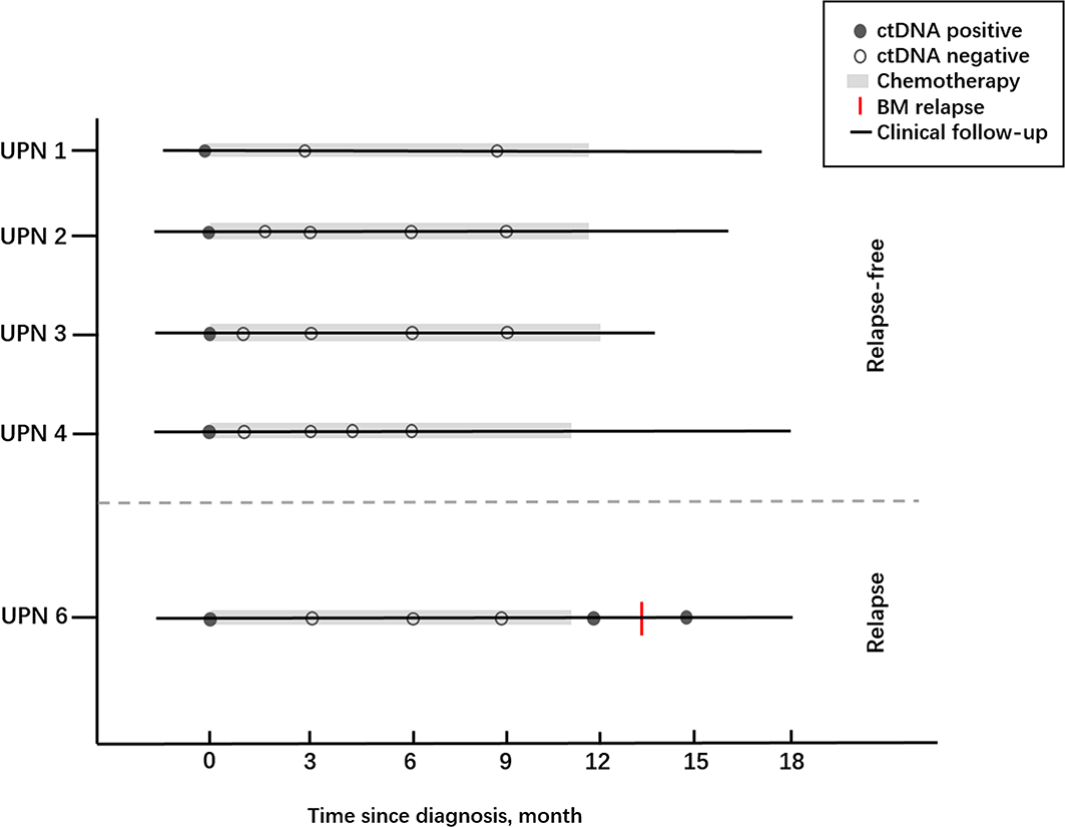
The clinical courses together with ctDNA statuses of five AML children who received chemotherapy.

To assess whether the dynamic change of ctDNA could reflect the status of MRD in AML patients, we investigated the concordance between the BM sample and ctDNA statuses of 5 out of 20 ctDNA-positive children at diagnosis (**Figure 9**). Of these five patients, one patient experienced relapse on the basis of the BM sample while four patient were relapse-free during the following surveillance. Notably, the results of serial plasma samples showed that four patients under the condition of relapse-free were with complete ctDNA clearance after chemotherapy and remained negative at the last follow-up. As for one patient who experienced relapse, ctDNA positivity regained after a temporary ctDNA clearance by chemotherapy, and recurrence of these cytogenetic abnormalities in ctDNA was observed nearly 2 months earlier than BM relapse. These results showed that ctDNA was basically consistent with the results from BM samples, and the shifting level of ctDNA may be a useful tool for MRD monitoring in children with AML.

## Discussion

In this study, we aim to evaluate the potential value of MRD based on positive ctDNA status in patients with AML, and the result reported that surveillance of matched serum ctDNA in residual driver mutation persistence may be regarded as an independent sample of MRD testing, which was comparable and with high concordance with sequencing of BM samples for the diagnosis of gene alterations in the AML children.

AML is a highly heterogeneous disease, and its diagnosis and treatment require a comprehensive analysis of morphology, immunology, genetics, and molecular biology. NGS, as a new molecular biological technology, has the advantages of high throughput, high sensitivity, and low cost and is an important means to explore the molecular pathogenesis of blood tumors and guide clinical diagnosis and treatment. Previously, detection of gene mutation by BM was the standard method to identify DNA aberration in AML patients. However, acquisition of the BM sample is traumatic, and it is usually difficult to collect specimens in succession for the close monitoring of MRD, which greatly limits its application in clinical practices. Moreover, the sensitivity of MRD monitoring from PB was much lower than that from BM (15, 16). This is true even under the circumstances of highly sensitive real-time PCR-based methods targeting leukemia-related gene alterations.

In 1948, Mandel and Metais firstly advanced the presence of cfDNA in human blood (17). Subsequently, Koffler et al. (18) found the higher concentration of cfDNA in the circulation of patients with cancer when compared with healthy people, indicating that the presence of the cancer patients may be simply screened through a test of PB. In 1994, cfDNA was regarded as an independent sample in distinguishing RAS mutations in patients with hematological oncology (19, 20). The recent introduction of NGS-based molecular approaches has further refined such MRD measurements with regard to broader applicability. ctDNA was a kind of noninvasive method that showed its great potential in identifying the gene mutation, and specifically for patients for whom no conventional genetic marker for MRD testing was available or conventional MRD approaches such as flow cytometry or cytogenetics were negative in AML children in recent years (21–24).

To date, data on the utility of ctDNA from PB in AML children are relatively sparse; moreover, the results regarding the diagnostic value in this population were still unspecified. In this study, it is the first time that ctDNA was used for the detection of genetics aberration in pediatric AML, and consistent results were found in this sample when compared with BM and PBMC samples on the distribution of targeted sites. Moreover, the absolute blast count in PB did not affect the result of ctDNA in identifying gene mutations. It was found that ctDNA has good consistency with BM in the analysis of mutation frequency, and ctDNA may identify some potential mutations that cannot be detected by NGS in the BM and PBMC sample.

MRD monitoring has been used as a vital tool for early prediction of the efficacy of chemotherapy in AML children. For MRD evaluation, the sample of choice is BM, although peripheral blood is easy to obtain and lacks immature normal populations of cells that may interfere with the analysis. ctDNA has the potential to capture intratumor heterogeneity that may be missed by BM analysis. In addition, ctDNA has an advantage of faster turn-around time as well as an acceptable running cost for serial monitoring of MRD. The current practice for the assessment of MRD for response assessments relies on BM sampling, whereas dynamic ctDNA monitoring may be adequate for reflecting the remission status in some AML cases. In view of this, these findings potentially introduce the utility of this noninvasive means at the time of diagnosis.

In summary, our results confirm that ctDNA may be used as a complementary method in reflecting the mutation spectrum and MRD monitoring of AML children, which may be particularly relevant in the context of subclonal mutations with lower VAF. However, these findings warranted a larger, prospective study to investigate the prognostic stratification and MRD monitoring in pediatric AML.

## Data Availability Statement

The datasets presented in this study can be found in online repositories. The names of the repository/repositories and accession number(s) can be found below: https://ngdc.cncb.ac.cn/gsa-human/browse/HRA000912.

## Ethics Statement

The study design and methods complied with the Declaration of Helsinki and was approved by the Ethics Committee and Institutional Review Board of Institute of Hematology and Blood Diseases Hospital, Chinese Academy of Medical Sciences & Peking Union Medical College. Informed consent was obtained from all subjects.

## Author Contributions

XZ and MR contributed to the idea, conception, and study design. MR and LpL wrote the manuscript and contributed to data analysis. BQ, FaL, SW, XL, YG, YZ, and YC collected, organized, and provided AML patient data. XyC, LC, LxL, CW, FeL, and SC conducted experiments, analyzed the data, and contributed to generating figures. AZ, XjC, and YcZ helped in writing and revising the manuscript, and generating the figures. LZ revised the manuscript and prepared it for submission.

## Funding

This work was supported by the CAMS Innovation Fund for Medical Sciences (CIFMS) (2020-I2M-C&T-B-087) and the National Natural Science Foundation of China (81500156, 81170470).

## Conflict of Interest

Author LxL, SC, FeL, and CW were employed by the company Acornmed Biotechnology Co., Ltd.

The remaining authors declare that the research was conducted in the absence of any commercial or financial relationships that could be construed as a potential conflict of interest.

## Publisher’s Note

All claims expressed in this article are solely those of the authors and do not necessarily represent those of their affiliated organizations, or those of the publisher, the editors and the reviewers. Any product that may be evaluated in this article, or claim that may be made by its manufacturer, is not guaranteed or endorsed by the publisher.
