# Supplementary material for: Targeted Next-Generation Sequencing of Circulating Tumor DNA, Bone Marrow, and Peripheral Blood Mononuclear Cells in Pediatric AML
Source: Front Oncol. 2021 Jul 29;11:666470. doi: 10.3389/fonc.2021.666470 (PMC8377768; doi:10.3389/fonc.2021.666470)
Supplement: Supplementary file 1 [file DataSheet_1.docx]

**Supplementary Tables and Figures**

Table S1: List of genes and target regions included in the customized version of the 137-gene panel

| Gene | Target region (exon) | Gene | Target region (exon) |
| --- | --- | --- | --- |
| ABL1 | exon 1-10 | KDM6A | exon 1-29 |
| ALK | all exons | KDM6B | exon 3-22 |
| ANKRD26 | all exons | KIT | exon 2, 7-17 |
| ARID1B | exon 1-19 | KMT2A | all exons |
| ASXL1 | exon 8-12 | KMT2C | exon 14, 43 |
| ASXL2 | all exons | KMT2D | all exons |
| ATM | all exons | KRAS | exon 1-4 |
| ATRX | exon 8-10, 17-35 | MACF1 | all exons |
| B2M | exon 1-2 | MAP2K1 | exon 1-3, 6 |
| BCL11B | all exons | MED12 | exon 1-2, 21 |
| BCL2 | all exons | MPL | exon 3-6, 10, 12 |
| BCL6 | all exons | MSH6 | all exons |
| BCOR | exon 1-14 | MYC | all exons |
| BCORL1 | all exons | MYD88 | all exons |
| BIRC3 | exon 2-9 | NF1 | all exons |
| BLM | exon 3-20 | NOTCH1 | exon 26-28, 34 |
| BRAF | exon 1-18 | NPM1 | exon 10-11 |
| BTK | exon 15 | NR3C1 | all exons |
| CACNA1G | all exons | NRAS | exon 1-3 |
| CALR | exon 8-9 | NSD2 | all exons |
| CBL | exon 8-9, 12, 16 | NT5C2 | exon 9-17 |
| CCDC168 | all exons | NUMB | all exons |
| CCND3 | all exons | PAX5 | all exons |
| CD79A | exon 4-5 | PCLO | all exons |
| CDKN1B | all exons | PDGFRA | exon 12, 14, 18 |
| CDKN2A | all exons | PDGFRB | all exons |
| CEBPA | all exons | PHF6 | exon 1-9 |
| CECR2 | all exons | PIGA | all exons |
| CREBBP | exon 25-28, 31 | PIK3R1 | all exons |
| CRLF2 | exon 6 | PLCG2 | exon 19-20, 24 |
| CSF3R | exon 12-17 | PPM1D | exon 6 |
| CTCF | all exons | PRKDC | all exons |
| CTLA4 | exon 1 | PRPF8 | exon 28 |
| CUX1 | all exons | PRPS1 | all exons |
| DDX41 | exon 5-6, 15 | PTEN | all exons |
| DHX15 | all exons | PTPN11 | exon 3, 12-13 |
| DHX30 | all exons | RAD21 | all exons |
| DIS3 | exon 10-11, 16 | RB1 | all exons |
| DKC1 | exon 1-14 | RPL10 | all exons |
| DNAH2 | all exons | RUNX1 | all exons |
| DNM2 | all exons | SETBP1 | exon 3-4 |
| DNMT3A | all exons | SETD2 | all exons |
| ELANE | exon 2-5 | SF3B1 | exon 13-16, 18 |
| EP300 | all exons | SH2B3 | all exons |
| EPOR | all exons | SMC1A | all exons |
| ETNK1 | exon 3 | SMC3 | all exons |
| ETV6 | all exons | SOS1 | all exons |
| EZH2 | all exons | SPI1 | all exons |
| FAM46C | exon 1 | SRCAP | all exons |
| FAT1 | all exons | SRP72 | exon 6-8 |
| FBXW7 | exon 8-12 | SRSF2 | all exons |
| FLT3 | exon 13-16, 20-21 | STAG2 | all exons |
| FOXO1 | exon 1-2 | STAT3 | exon 21 |
| GART | all exons | STAT5B | exon 14-16 |
| GATA1 | exon 2 | TERC | exon 1 |
| GATA2 | exon 2-6 | TERT | exon 1-16 |
| GATA3 | exon 2-6 | TET2 | all exons |
| GFI1 | exon 7 | TNFAIP3 | exon 2-9 |
| GNAS | exon 1, 8-9 | TNFRSF14 | all exons |
| HAX1 | exon 2-3 | TP53 | exon 2-11 |
| ID3 | all exons | TPMT | exon 3-6, 8-9 |
| IDH1 | exon 2, 4 | TRIM24 | all exons |
| IDH2 | exon 4-5 | U2AF1 | exon 2, 6-7 |
| IKZF1 | all exons | USH2A | all exons |
| IL7R | exon 5-6 | USP7 | all exons |
| JAK1 | exon 9-18 | VEGFC | all exons |
| JAK2 | exon 7-25 | WT1 | all exons |
| JAK3 | exon 3-20 | ZRSR2 | all exons |
| KDM5C | all exons |  |  |

Figure S1 Mutation of bone marrow (BM) and ctDNA on basis of next generation sequencing (NGS) in 20 patients. On the left is the sample source of the checkout point. The light blue region is the ctDNA specific detection site, the dark blue region is the BM specific detection site, and the gray region is the mutation found by BM and ctDNA at the same time. On the right is the total number of loci detected in each patient.

Figure S2 A: Consistency of SNV frequency between BM and ctDNA.

B: Consistency of Indel frequency between BM and ctDNA.

Figure S3. Frequency distribution of BM and ctDNA specific sites.

Figure S4. Aberrant mutation of clinical significance detected by BM, PBMC and ctDNA.

Figure S5 Display of clinical significance site - a map of patient mutations with specific sites. The horizontal axis shows the clinical significance site, and the vertical axis shows the mutation frequency of this site in each sample type. Red represents bone marrow detection, black represents PBMC detection, and blue represents ctDNA detection.
