# Supplementary figures and images for: Targeted Next-Generation Sequencing of Circulating Tumor DNA, Bone Marrow, and Peripheral Blood Mononuclear Cells in Pediatric AML
Source: Front Oncol. 2021 Jul 29;11:666470. doi: 10.3389/fonc.2021.666470 (PMC8377768; doi:10.3389/fonc.2021.666470)

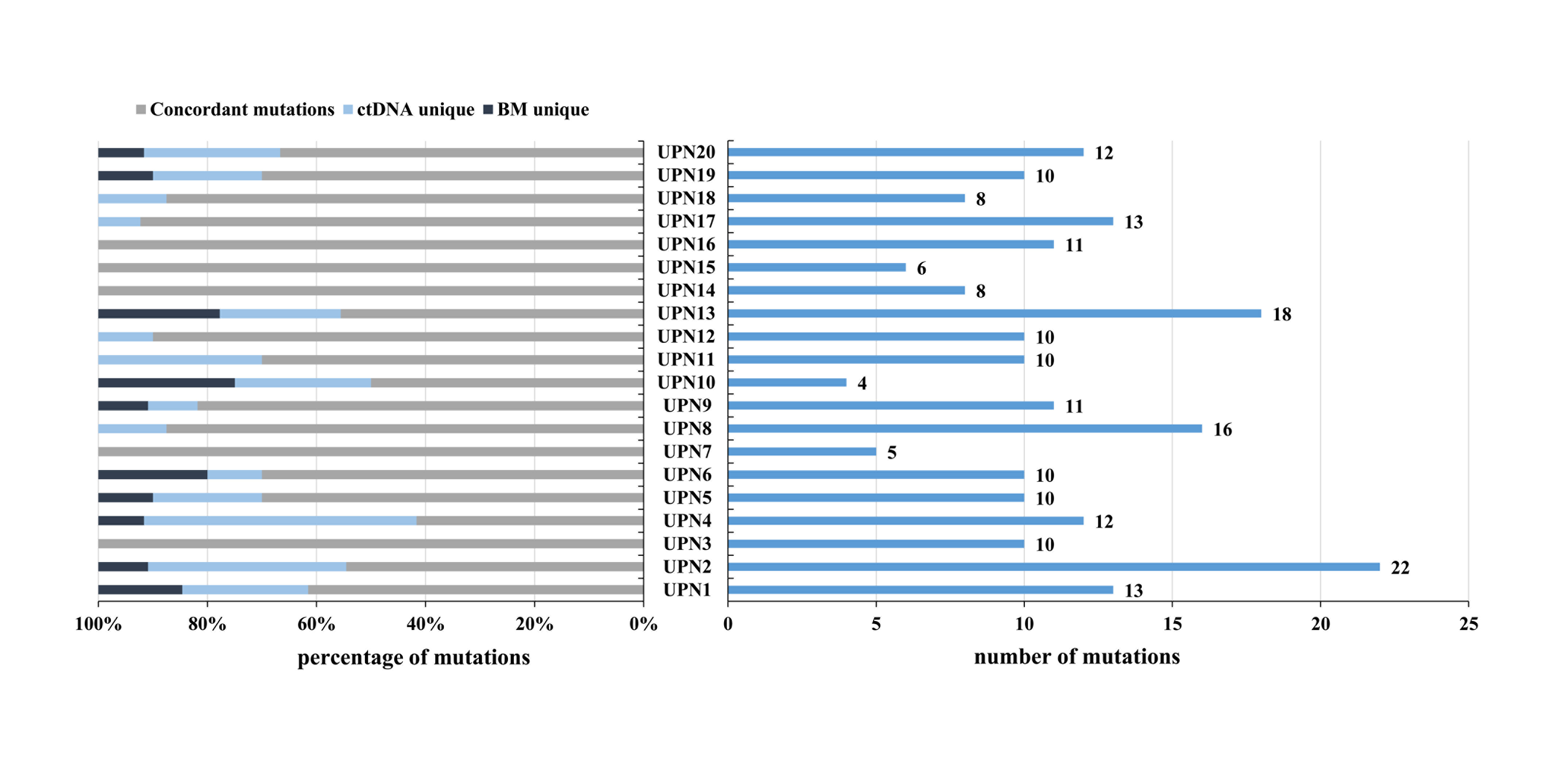

Supplement: Supplementary file 2 [file Image_1.tif]

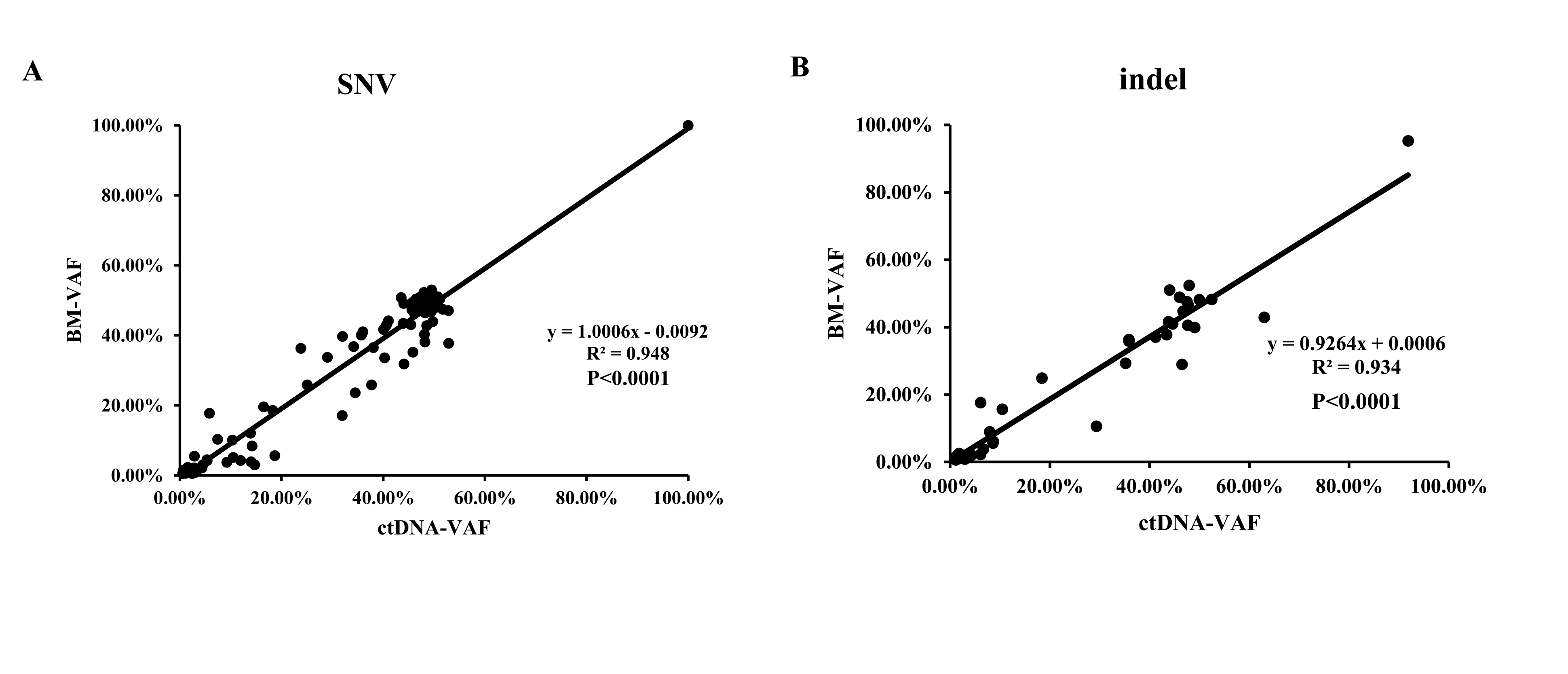

Supplement: Supplementary file 3 [file Image_2.jpeg]

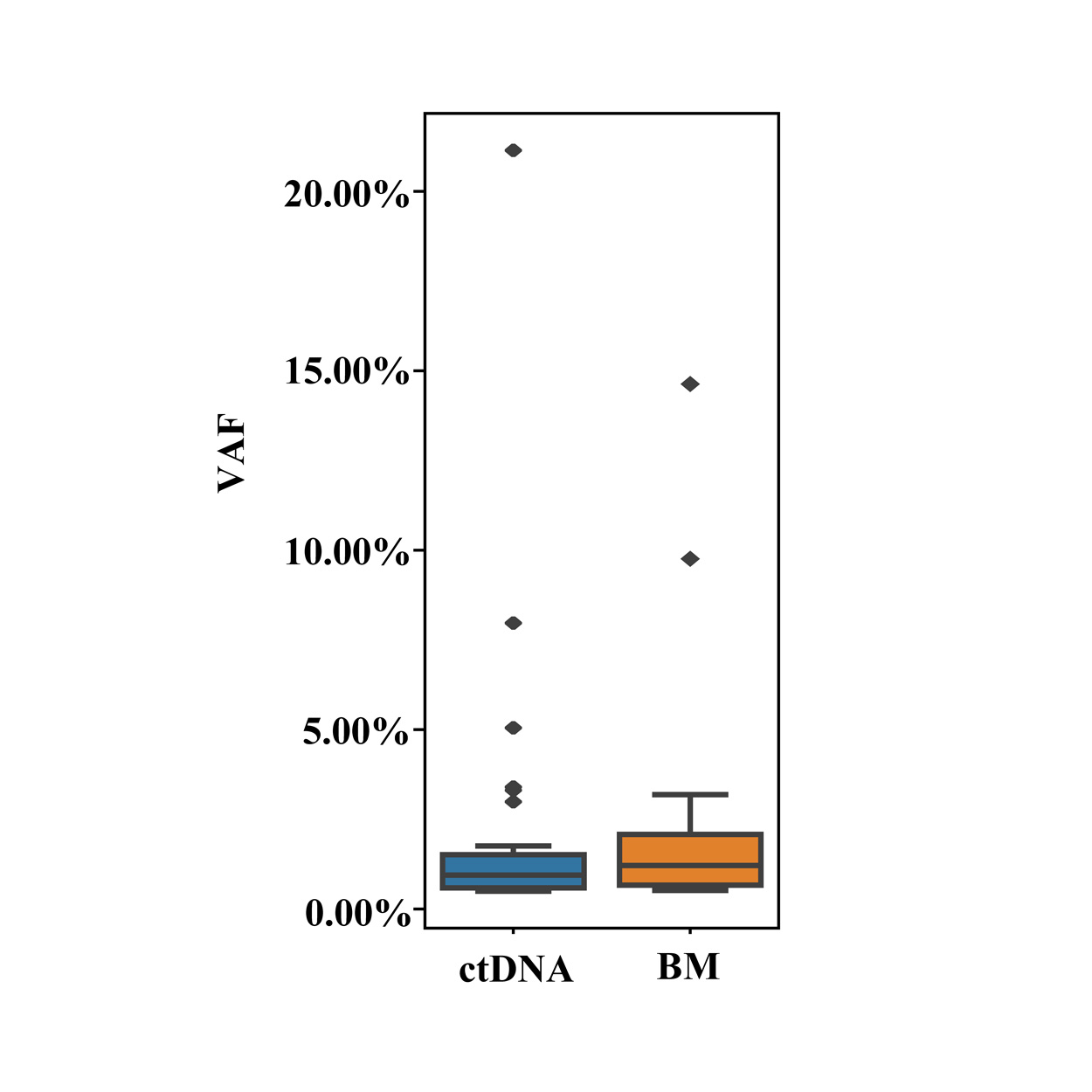

Supplement: Supplementary file 4 [file Image_3.tif]

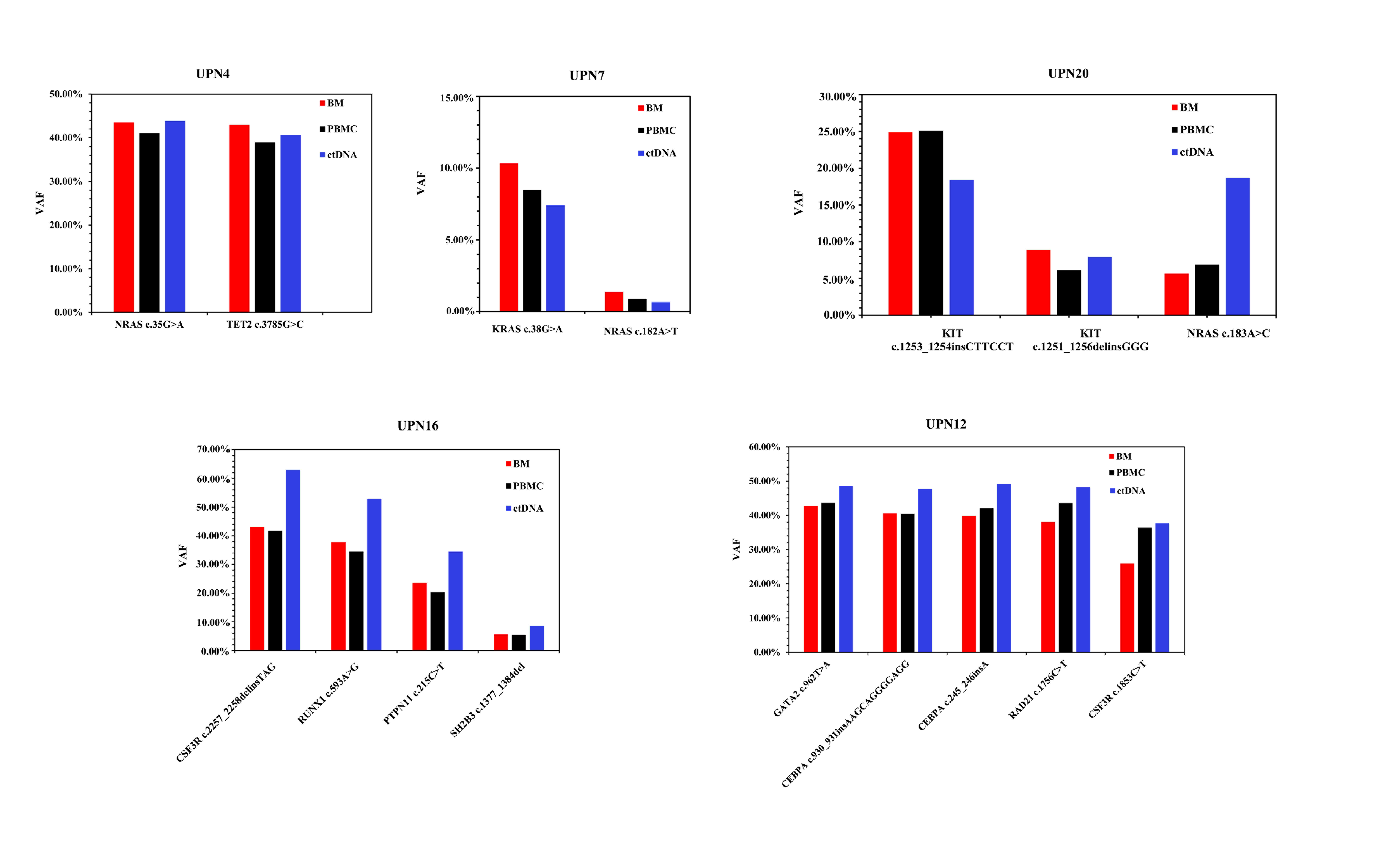

Supplement: Supplementary file 5 [file Image_4.tif]

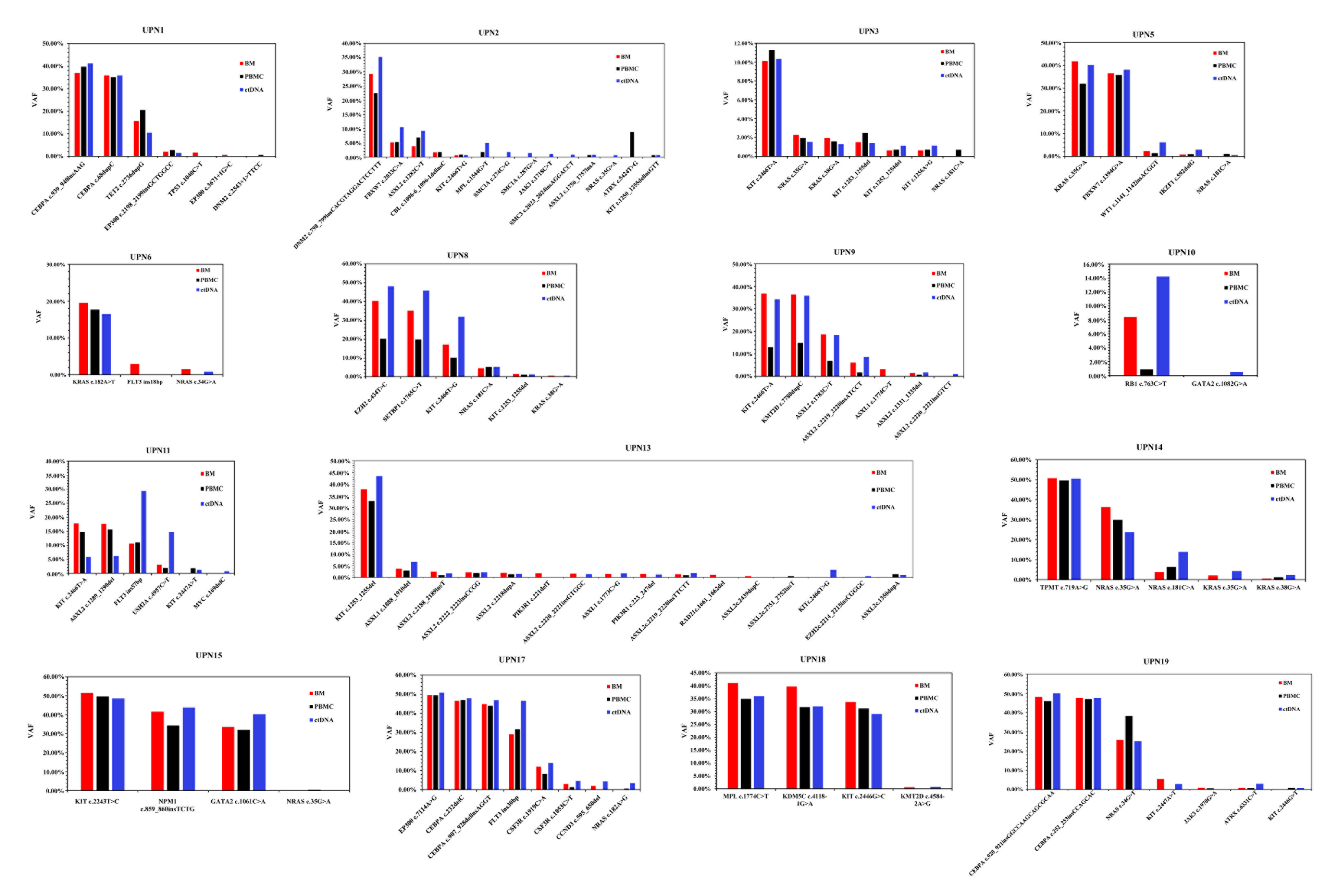

Supplement: Supplementary file 6 [file Image_5.tif]
